# QT prolongation in the STREAM Stage 1 Trial

**DOI:** 10.5588/ijtld.21.0403

**Published:** 2022-04-01

**Authors:** G. Hughes, H. Bern, C-Y. Chiang, R. L. Goodall, A. J. Nunn, I. D. Rusen, S. K. Meredith

**Affiliations:** 1 Medical Research Council Clinical Trials Unit at University College London, Institute of Clinical Trials and Methodology, London, UK; 2 Division of Pulmonary Medicine, Department of Internal Medicine, Wanfang Hospital, Taipei Medical University, Taipei, Taiwan; 3 Division of Pulmonary Medicine, Department of Internal Medicine, School of Medicine, College of Medicine, Taipei Medical University, Taipei, Taiwan; 4 International Union Against Tuberculosis and Lung Disease (The Union), Paris, France; 5 Research Division, Vital Strategies, New York, NY, USA

**Keywords:** rifampicin-resistant tuberculosis, moxifloxacin, clofazimine, QTcF, electrocardiogram

## Abstract

**BACKGROUND:**

STREAM (Standardized Treatment Regimen of Anti-TB Drugs for Patients with MDR-TB) Stage 1 demonstrated non-inferior efficacy of a shortened regimen (the Short regimen) for rifampicinresistant TB (RR-TB) compared to the contemporaneous WHO-recommended regimen. This regimen included moxifloxacin and clofazimine, known to cause QT prolongation, and severe prolongation was more common on the Short regimen. Here we investigate risk factors for QT prolongation with the Short regimen.

**METHODS:**

Data from patients prescribed the Short regimen (*n* = 282) were analysed to identify risk factors for severe QT prolongation (QT/QTcF ≥500 ms or ≥60 ms increase in QTcF from baseline).

**RESULTS:**

Of the 282 patients on the Short regimen, 94 (33.3%) developed severe QT prolongation: 31 QT/QTcF ≥500 ms; 92 experienced ≥60 ms QTcF increase from baseline. The median time to QT/QTcF ≥500 ms was 20 weeks (IQR 8–28), and the time to ≥60 ms increase from baseline was 18 weeks (IQR 8–28). Prolongation ≥500 ms was most frequent in patients from Mongolia (10/22, 45.5%) compared with 3.5–11.9% at other sites, *P* < 0.001. Higher baseline QTcF increased risk of prolongation to ≥500 ms (QTcF ≥400 ms: OR 5.99, 95% CI 2.04–17.62).

**CONCLUSION:**

One third of patients on the Short regimen developed severe QT prolongation. QT/QTcF ≥500 ms was more common in patients from Mongolia and in those with a higher baseline QTcF, which may have implications for implementation of treatment.

## Abstract (fr)

**CONTEXTE:**

L’essai clinique STREAM (*Standardized Treatment Regimen of Anti-TB Drugs for Patients with MDR-TB*)Étape 1 a démontré l’efficacité non inférieure d’un schéma plus court pour le traitement de la TB résistante à la rifampicine (RR-TB) par rapport au schéma recommandé par l’OMS. Ce schéma comprenait moxifloxacine et clofazimine, connues pour provoquer un allongement de l’intervalle QT. Les cas graves d’allongement de l’intervalle QT étaient plus fréquents sous schéma court. Nous avons analysé les facteurs de risque d’allongement de l’intervalle QT chez les patients sous schéma court.

**MÉTHODES:**

Les données des patients randomisés sous schéma court (n = 282) ont été analysées pour identifier les facteurs de risque de grave allongement de l’intervalle QT (QT/QTcF ≥500 ms ou augmentation ≥60 ms de l’intervalle QTcF par rapport à l’inclusion).

**RÉSULTATS:**

Ont été associés 94 (33,3%) patients sous schéma court à un grave allongement de l’intervalle QT : 31 cas de QT/QTcF ≥500 ms ; 92 cas d’augmentation ≥60 ms de l’intervalle QTcF par rapport à l’inclusion. Le temps médian avant QT/QTcF ≥500 ms était de 20 (IQR 8–28) semaines et de 18 semaines (IQR 8–28) avant augmentation ≥60 ms par rapport à l’inclusion. Un allongement ≥500 ms était plus fréquent chez les patients de Mongolie (10/22, 45,5%), par rapport à 3,5–11,9% pour les autres pays (*P* < 0,001). Un intervalle QTcF plus élevé à l’inclusion augmentait le risque d’allongement ≥500 ms (QTcF ≥400 ms : OR 5,99, IC 95% 2,04–17,62).

**CONCLUSION:**

Un tiers des patients sous schéma court ont été associés à un grave allongement de l’intervalle QT. Un intervalle QT/QTcF ≥500 ms était plus fréquent chez les patients de Mongolie et chez ceux dont l’intervalle QTcF était plus élevé à l’inclusion, ce qui pourrait impacter l’instauration du traitement.

The treatment of drug-resistant TB has improved over recent years with the development of shorter and more effective regimens. There may, however, be a trade-off between treatment improvement and risk of adverse events.^1^ The STREAM Stage 1 Trial was a Phase 3 non-inferiority randomised controlled trial for patients with pulmonary rifampicin-resistant TB (RR-TB), which demonstrated that a “short” 9-month regimen was non-inferior to a “long” 20-month regimen that was standard of care.^2^,^3^

Patients were randomised in a 2:1 ratio in favour of the Short regimen which was based on the regimen studied in Bangladesh,^4^ replacing gatifloxacin with moxifloxacin (MFX), as the product was no longer available to good manufacturing practice standards.^5^ Patients were recruited from four countries (Ethiopia, Mongolia, South Africa and Vietnam) between 2012 to 2015; 424 were randomised—282 to the Short regimen and 142 to the Long regimen.

The Short regimen (details in Table 1) includes MFX and clofazimine (CFZ), both known to independently cause QT prolongation; in addition, high-dose MFX was used to reduce the development of resistance and improve efficacy.^6^ Although all fluoroquinolones may affect QT, MFX has been shown to have the greatest effect,^7^ which is more pronounced at higher doses.^8^ CFZ has also been associated with QT prolongation^9^,^10^ and arrhythmia torsades de pointes (TdP).^11^ The QT prolongation effect of these two drugs is thought to result from the inhibition of the *hERG* potassium channel.^12^,^13^ Particular attention to cardiac safety in the trial was therefore warranted. Patients prescribed the Long regimen received a standard dose of MFX or levofloxacin (LVX), both of which are known to prolong the QT interval, although this risk was considered lower.^14^–^16^ Frequent electrocardiogram (ECG) monitoring was undertaken in all trial patients up to Week 52.

**Table 1:** Drugs and doses given in the Short regimen

Phase and duration	Drug	Weight group		

		<33 kg	33–50 kg	>50 kg
Intensive and continuation (40 weeks)	Moxifloxacin	400 mg	600 mg	800 mg
	Clofazimine	50 mg	100 mg	100 mg
	Ethambutol	800 mg	800 mg	1,200 mg
	Pyrazinamide	1,000 mg	1,500 mg	2,000 mg
Intensive (16 weeks)	Isoniazid	300 mg	400 mg	600 mg
	Prothionamide	250 mg	500 mg	750 mg
	Kanamycin^*^	15 mg per kg body weight (maximum 1 g)		

^*^ Kanamycin given three times weekly after 12 weeks.

Patients developing a QT or QTcF ≥500 ms are at increased risk of tachyarrythmias such as TdP; this risk is lower in patients who experience ≥60ms increase in QTcF over baseline.^17^ It is estimated that there is a 5% increased risk of arrhythmia for every 10 ms increase in QT interval above the upper limit of normal.^18^ Patients with a corrected QT (QTc) interval ≥500 ms during screening were ineligible for trial inclusion.

## Electrocardiogram monitoring

A baseline 12-lead ECG was performed at enrolment and repeated 4 h after the first dose of trial medication. ECGs were initially performed weekly until Week 4 and then at 12, 24 and 36 weeks. The protocol was amended after 18 months (January 2014) to require ECGs every 4 weeks from Week 4 to Week 52. We used Fredericia’s formula (QTcF) to correct the QT interval for patients’ heart rate, as recommended in US Food and Drug Administration guidance.^19^ All sites in the study used a MAC 800 (GE Healthcare, Chicago, IL, USA) machine with a paper speed of 25 mm/s and voltage gain of 10 mm/mV. All machines were calibrated at baseline and the machine estimate of the QT and QTcF interval were recorded.

Analysis of all patients in STREAM Stage 1 who received at least one dose of trial medication (safety analysis population) showed a higher number of patients exceeding a maximum QT or QTcF of ≥500 ms after start of treatment in the Short regimen than in the Long regimen (11% vs. 6.4%; *P* = 0.14).^2^

If a patient had QT or QTcF prolongation ≥500 ms, treatment was reviewed and adjusted if required. No treatment changes were advised because of an increase QTcF of ≥60 ms from baseline value alone. Both are considered a DAIDS (Division of AIDS) Grade 3 event. Of the 31 (11%) patients who received the Short regimen and developed a QT or QTcF prolongation ≥500 ms, 7 continued treatment with no modifications, 4 had a permanent dose reduction of MFX alone, 20 had either MFX and/or CFZ treatment interruption, dose reduction and/or a change of drug; MFX was replaced by LVX in 11 patients and 3 patients permanently discontinued CFZ.^2^

TdP was not observed in 12-lead ECG recordings for any patient. Four cases of sudden death occurred at home during treatment (three on the Short regimen; one on the Long regimen), although only two were attributed to anti-TB treatment (one on each regimen) at independent review of deaths. In two of the cases on the Short regimen, a QT or QTcF of ≥500ms and an increase in QTcF ≥60 ms from baseline had been recorded.

The objective of this paper is to describe the evolution of QT prolongation on treatment over time in both the Short and Long regimens and to identify factors predictive of increased risk of developing QT or QTcF of ≥500 ms or ≥60 ms QTcF increase from baseline on the Short regimen.

## METHODS

The Short regimen is shown in Table 1. The intensive phase could be extended by up to 8 weeks if smear remained positive at 16 or 20 weeks of treatment. Patients on the Long regimen were given medication according to their country’s National Tuberculosis Programme (NTP). The intensive phase could be extended beyond 8 months if the smear remained positive, consistent with WHO 2011 recommendations.^3^ No Long regimen patients were given CFZ, although they all received standard-dose MFX or LVX.

Known risk factors for QT prolongation such as hypokalaemia, and renal and hepatic impairment were monitored with routine bloods collected monthly during the intensive phase. Thyroid function, hypomagnesaemia and hypocalcaemia were not routinely checked.

### Statistical analysis

A retrospective analysis was undertaken of QT and QTcF intervals during follow-up. Statistical analyses were conducted using STATA v15.1 (Stata, College Station, TX, USA).

The timing and value of the maximum QTor QTcF was identified for each patient, with ECG data censored at the point two or more drug changes occurred, indicating the patient was no longer taking the allocated regimen. The cumulative probability of maximum QT/QTcF exceeding 500 ms over time was estimated using a Kaplan–Meier curve and compared between treatment arms using a log-rank test. The effect of treatment arm on the hazard of maximum QT/QTcF exceeding 500 ms was estimated using a Cox proportional hazards model.

Patients assigned to the Short regimen arm were classified according to whether or not they had experienced severe QT prolongation at any time up to their Week 52 visit.

Univariable comparisons of baseline characteristics between patients with and without severe QT prolongation were conducted using the χ^2^ or Fisher’s exact tests for categorical variables and *t*-tests or Mann–Whitney rank tests for continuous variables. Those comparisons found to be significantly associated with severe QT prolongation at the 10% level were included in a multivariable logistic regression model in addition to the a priori factors of age, baseline QTcF reading and baseline potassium levels. Backwards elimination, with exit probability *P* = 0.05, was employed to select the final model. The association between HIV status and severe QT prolongation was explored in analyses restricted to countries, with at least 5% of participants being HIV-positive (South Africa and Ethiopia, which together had 98% of HIV-positive patients in the trial).

### Ethical considerations

The Ethics Advisory Group of the International Union Against Tuberculosis and Lung Disease (The Union, and its North American affiliate, the trial sponsor), Paris, France, and all relevant national and local ethics committees approved the trial. Informed consent was sought for the original study. As this study describes secondary analyses of trial data, further informed consent was not sought.

## RESULTS

### Evolution of QT/QTcF prolongation over time by regimen

**Figure 1:**
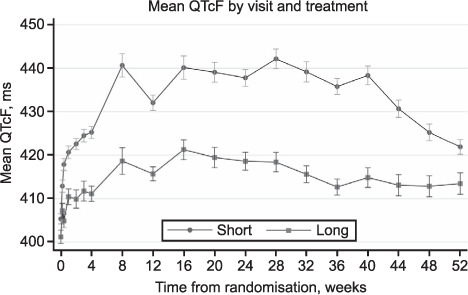
Mean QTcF by visit and allocated treatment arm.

**Figure 2:**
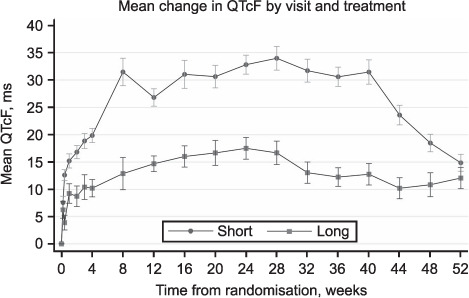
Mean change in QTcF from baseline by visit and allocated treatment arm. Mean QTcF and mean change in QTcF presented with error bars detailing ± 1 standard error of the mean.

Figure 1 shows the mean and standard error of the QTcF by visit and treatment arm. The difference in patient numbers assessed at each visit are largely explained by the protocol-mandated ECG monitoring in place at the time (Table 2). By Week 4, there is a clear separation between the two arms which increased at Week 8, after which time there is little change until Week 40, when the QTcF on the Short regimen declined, reflecting the completion of treatment for most patients on that regimen. Patients who received the Long regimen experienced a median maximum change in QTcF from baseline of 30 ms (IQR 22–41) compared to 50 ms (IQR 36–65) on the Short regimen. A maximum difference of 24 ms between the two arms was seen at Week 28. A similar pattern was seen for the mean change in QTcF from baseline (Figure 2).

**Table 2:** Number of participants with ECG reading across visits by treatment arm

	Number of participants with ECG reading across visits by treatment arm																		

	Baseline	2 h	4 h	Week 1	Week 2	Week 3	Week 4	Week 8	Week 12	Week 16	Week 20	Week 24	Week 28	Week 32	Week 36	Week 40	Week 44	Week 48	Week 52
Short regimen	282	280	280	270	272	270	267	152	259	155	160	253	180	188	244	198	192	193	194
Long regimen	141	138	138	137	136	136	137	72	130	76	77	128	86	89	127	95	88	96	97

ECG = electrocardiogram.

**Figure 3:**
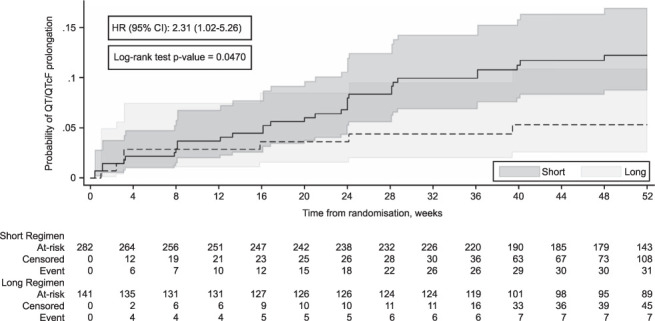
Kaplan–Meier plot of time to exceeding maximum QT/QTcF of 500 ms post baseline.

Thirty-one (11%) participants on the Short regimen reached a maximum QT/QTcF of 500 ms vs. seven (5%) on the Long regimen up until 52 weeks of follow-up with routine ECG monitoring. We observed a significant difference between treatment regimens in time to QT/QTcF ≥500 ms (*P* = 0.047; Figure 3); participants taking the Short regimen had a higher risk of reaching a maximum QT/QTcF ≥500 ms (hazard risk 2.31, 95% confidence interval [CI] 1.02–5.26) (Figure 3).

### Factors predictive of QT prolongation on the Short regimen

Of the 282 participants on the Short regimen, 94 (33.3%) had severe QT prolongation on at least one ECG; 31 participants had QT/QTcF of ≥500 ms, at a median time of 20 weeks (IQR 8–28) and 92 had ≥60 ms increase in QTcF from baseline at a median time of 18 weeks (IQR 8–28). The median QTcF change was 102 ms (IQR 81–137) in those with QT/QTcF ≥500 ms and 72 ms (IQR 66–94) in those with a QTcF ≥60 ms increase from baseline.

No statistically significant association (*P* > 0.1) was observed between severe QT prolongation and sex or baseline smoking status, diabetes, height, weight, body mass index, MFX and CFZ dose, liver function tests, glucose or potassium (Supplementary Tables S4 and S5). Analyses restricted to South African and Ethiopian participants showed no significant association between HIV status and severe QT prolongation.

A significant association between both country and baseline QTcF with the development of QT prolongation ≥500 ms remained after adjustment for other variables (Table 3). Mongolia showed the greatest association (10/22, 45.5%) compared with 3.5–11.9% of patients at the other sites (*P* < 0.001); baseline QTcF ≥400 ms was associated with increased risk compared to <400 ms (odds ratio 5.99, 95% CI 2.04–17.62).

**Table 3:** Univariable and multivariable analysis of risk factors for a QT/QTcF ≥500 ms in the Short regimen arm

		Patients with QT/QTcF ≥500 ms at any point, %	*n/N*	Univariable analysis OR (95% CI)	*P* value	Multivariable analysis aOR (95% CI)	*P* value
Baseline QTcF, ms	0–399	3.3	4/122	1.00 (base)	0.001	1.00 (base)	0.007
	≥400	16.9	27/160	5.99 (2.04–17.62)	—	4.77 (1.54–14.71)	—
Age (10 additional years)	—	—	—	1.51 (1.09–2.09)	0.013	1.43 (0.98–2.08)	0.063
Age category, years	18–24	4.7	3/64	1.00 (base)	0.092	—	—
	25–34	8.4	8/95	1.87 (0.48–7.33)	—	—	—
	35–44	14.9	10/67	3.57 (0.93–13.62)	—	—	—
	≥45	17.9	10/56	4.42 (1.15–16.98)	—	—	—
Baseline potassium, mmol/L	≥3.5	11.2	28/249	1.00 (base)	0.614	1.00 (base)	0.353
	<3.5	15.0	3/20	1.39 (0.38–5.05)	—	1.98 (0.47–8.34)	—
Country	Ethiopia	3.5	3/85	1.00 (base)	<0.001	1.00 (base)	<0.001
	Vietnam	7.6	5/66	2.24 (0.52–9.74)	—	1.01 (0.21–4.76)	—
	South Africa	11.9	13/109	3.70 (1.02–13.44)	—	2.48 (0.65–9.52)	—
	Mongolia	45.5	10/22	22.78 (5.48–94.74)	—	15.45 (3.45–69.24)	—
Baseline creatinine (additional 10 μmol/L)	—	—	—	0.78 (0.63–0.97)	0.022	—	—

OR = odds ratio; CI = confidence interval; aOR = adjusted OR.

There was some evidence of an association with increased risk of QT/QTcF ≥500 ms for participants with lower baseline creatinine levels and increased age on univariable analysis, but not after adjustment for other factors in the multivariable analysis. There was no evidence that baseline hypokalaemia increased the risk of severe QT prolongation.

Of the factors explored, only country was significantly associated with an increase in QTcF of ≥60 ms from baseline on univariable analysis, with Mongolia having a significantly higher proportion of cases (*P* < 0.001; Table 4); the country remained independently associated with QTcF increase in the multivariable analysis. In addition, there was evidence suggesting that a lower baseline QTcF interval significantly raised the risk of having an increase in QTcF of ≥60 ms from baseline. Age did not appear to be associated with risk of developing a ≥60 ms increase in QTcF from baseline.

**Table 4:** Univariable and multivariable analysis of risk factors for a QTcF ≥60 ms increase from baseline in the Short regimen arm

		Patients with QTcF ≥60 ms from baseline %	*n/N*	Univariable analysis OR (95% CI)	*P* value	Multivariable analysis aOR (95% CI)	*P* value
Baseline QTcF, ms	0–399	37.7	46/122	1.00 (base)	0.113	1.00 (base)	0.014
	≥400	28.7	46/160	0.67 (0.40–1.10)	—	0.49 (0.27–0.87)	—
Age (10 additional years)	—	—	—	1.11 (0.89–1.39)	0.352	1.02 (0.79–1.33)	0.880
Age category, years	18–24	34.4	22/64	1.00 (base)	0.373	—	—
	25–34	27.4	26/95	0.72 (0.36–1.43)	—	—	—
	35–44	31.3	21/67	0.87 (0.42–1.81)	—	—	—
	45+	41.1	23/56	1.33 (0.63–2.79)	—	—	—
Baseline potassium, mmol/L	≥3.5	32.5	81/249	1.00 (base)	0.496	1.00 (base)	0.554
	<3.5	40.0	8/20	1.38 (0.54–3.52)	—	1.36 (0.49–3.74)	—
Country	Ethiopia	20.0	17/85	1.00 (base)	<0.001	1.00 (base)	<0.001
	Vietnam	40.9	27/66	2.77 (1.34–5.71)	—	3.17 (1.40–7.15)	—
	South Africa	28.4	31/109	1.59 (0.81–3.12)	—	1.74 (0.84–3.63)	—
	Mongolia	77.3	17/22	13.60 (4.39–42.10)	—	22.86 (6.42–81.40)	—

OR = odds ratio; CI = confidence interval; aOR = adjusted OR.

Just over half the patients who developed severe QT prolongation were taking the higher 800 mg dose of MFX (18/30 participants who developed QT/QTcF ≥500 ms and 47/92 participants who had ≥60 ms QTcF increase from baseline). MFX and CFZ dose at baseline (in mg/kg) was not found to significantly increase the risk of severe QT prolongation (Supplementary Figure S1, Supplementary Tables S4 and S5). Mean baseline MFX was 13.7 mg/kg (standard deviation [SD] 1.4) in those who did not develop severe QT prolongation vs. 13.5 mg/kg (SD 1.5) in those with QT/QTcF ≥500 ms. Mean baseline CFZ was 2.0 mg/kg (SD 0.3) in the no prolongation group vs. 1.9 mg/kg (SD 0.4) in the QT/QTcF ≥500 ms group. This was similar for participants with ≥60 ms QTcF increase from baseline.

To further investigate the effect of MFX and CFZ dosing per kg, patients were separated by weight band (33–50 kg and >50 kg, administered MFX 600 mg and 800 mg, respectively). Patients weighing 33–50 kg who developed QT/QTcF ≥500 ms (*n* = 13) received higher mg/kg doses of both drugs at Weeks 8, 12 and 16 than those who did not (*P* ≤ 0.05 for all three time points). However, this relationship was not found in the >50 kg weight band (Supplementary Tables S1–S3). Although total drug dose remained constant through these time points, patient weight changed, affecting the mg/kg dose. No difference in *P* values was seen for MFX or CFZ at the same time point, as total drug dose remained constant (Supplementary Tables S2 and S3).

No association was observed between mg/kg dose of MFX or CFZ and ≥60 ms QTcF increase from baseline, either when weight bands were combined or separated (data not shown).

## DISCUSSION

The evolution of QTcF over time showed a clear difference between regimens after the first month of treatment. Participants administered the Short regimen experienced a greater increase in QTcF than those on the Long regimen. Mean QTcF values fell sharply after treatment completion at Week 40 on the Short regimen; this suggests that MFX has a much shorter half-life than CFZ,^20^,^21^ which plays a key role in QT prolongation. However, the increase in QTcF values at the end of treatment compared to baseline may be due to persistence of CFZ. It is difficult to disentangle the separate effects of MFX and CFZ on QT prolongation in this analysis. The small increase in QTcF with the Long regimen that remained at Week 52 when patients were still on treatment is likely to be due to the standard-dose fluoroquinolone in regimen. It is also possible that other drugs contributed to QT prolongation through secondary adverse effects such as hypothyroidism with prothionamide and renal dysfunction with kanamycin,^1^ which were used in the Long regimen.

We found that risk factors for the development of QT/QTcF ≥500 ms on the Short regimen included being from the Mongolian site and having a baseline QTcF ≥400 ms. The latter is not surprising since such patients were closer to the threshold of 500 ms. Case reports of LVX-induced QT prolongation and TdP have found patients had a high baseline value,^22^,^23^ and a review of 900 patients admitted to a cardiac care unit found a baseline QTc >450 ms was an independent risk factor for severe QTc prolongation.^24^

The increased risk of severe QT prolongation (both development of QT/QTcF ≥500 ms and ≥60 ms QTcF increase from baseline) in patients from the Mongolian site was unexpected, and may represent genetic or environmental differences that affected the pharmacokinetics (PK) of the trial medications. Genes such as *KCNQ1* and *SCN5A* (associated with congenital long QT syndrome) have been described in Mongolia and the relative genetic isolation has been proposed as a possible cause for QT prolongation in this population.^25^–^29^ Single nucleotide polymorphisms in the *UGT1A* gene are known to affect the metabolism of MFX.^30^ The absence of increased risk in the Vietnamese patients in STREAM Stage 1 would support regional variation among Asian populations. Among trial sites, hypothermia (a known cause of QT prolongation) was a risk specific to Mongolia with winter temperatures averaging −15° to −30°C, which may have affected some patients.^31^

While kanamycin can cause QT prolongation indirectly through renal impairment and electrolyte abnormalities,^1^ our analysis showed a lower mean creatinine at baseline in patients who developed QT/QTcF ≥500 ms on the Short regimen. However, this was not seen at later time points and does not seem clinically plausible. The apparent lack of association between hypokalaemia and risk of QT prolongation was unexpected. However, our ability to examine the relationship was limited by lack of testing, except during the intensive phase.

Increasing age is associated with QT prolongation,^32^ but although we found some evidence of association between age and QT/QTcF ≥500 ms in univariable analysis, this was not maintained in the multivariable model and not seen in relation to risk of ≥60 ms QTcF increase from baseline. However, our population was relatively young, with most participants less than 45 years of age.

High-dose MFX, in combination with CFZ in the Short regimen, is likely to explain the differences in QT prolongation between regimens; however, the lack of relationship between weight-adjusted dose (mg/kg) and maximum QTcF was surprising. Although we found that those administered MFX 600 mg who developed QT/QTcF ≥500 ms were more likely to receive a higher mg/kg dose; this was not seen in the higher weight band >50 kg who were administered MFX 800 mg.

This study had limitations. First, this is a post-hoc analysis of the trial data. Second, safety bloods were only recorded routinely during the intensive phase. It is possible that risk factors for QT prolongation such as hypokalaemia and hypothyroidism, had been prevalent during the continuation phase. Third, no PK studies were undertaken in STREAM Stage 1, which may have enlightened the relationship between weight-adjusted dose and maximum QTcF. This study has a number of strengths. First, the QT data presented are from routine ECG monitoring that took place at the same time point for all patients and was not modified by the clinical situation. Second, participants were randomised from multiple sites in several different countries and were from different ethnic groups; this adds weight to the generalisability of the findings. Third, this is the largest analysis to date of QT prolongation in the Short regimen from a randomised controlled trial.

The majority of patients on the Short regimen did not experience severe QT prolongation, and higher-dose fluoroquinolone in combination with CFZ could be used safely in most patients. As patients with baseline QTcF ≥400 ms appeared to be at greater risk, they may need closer monitoring in programmatic settings. Most patients who reached the 500 ms threshold did so ≥3 months after start of treatment, suggesting ECG monitoring may be required throughout treatment with this regimen. Fluoroquinolones and CFZ are now frequently used in regimens alongside bedaquiline and/or delamanid, such as in the WHO-recommended all-oral regimen.^33^ his could potentiate the QT prolongation seen in STREAM Stage 1.^34^,^35^ The results from STREAM Stage 2 will provide important information on the safety of an all-oral regimen.
